# Comparative study on the biosynthesis of magnetite nanoparticles using *Aspergillus elegans* extract and their efficacy in dye degradation versus commercial magnetite nanoparticles

**DOI:** 10.1016/j.heliyon.2024.e40747

**Published:** 2024-11-28

**Authors:** Azhin H. Mohammed, Renjbar M. Mhammedsharif, Parwin J. Jalil, Sida M. Mhammedsharif, Ahmed S. Mohammed

**Affiliations:** a Physics Department, College of Education, University of Sulaimani, Kurdistan Region, Iraq; b Scientific Research Centre, Soran University, Kurdistan Region, Iraq; c Civil Engineering Department, College of Engineering, University of Sulaimani, Kurdistan Region, Iraq

**Keywords:** *Aspergillus elegans*, Magnetite nanoparticle, Characterization, Catalytic activity

## Abstract

This study compares magnetite (Fe3O4) nanoparticles synthesized using Aspergillus elegans extract versus commercially available magnetite nanoparticles, focusing on their efficacy in dye degradation. The biosynthesis of Fe3O4 nanoparticles using fungal extracts offers a sustainable and eco-friendly alternative to conventional chemical methods. The nanoparticles were characterized using various techniques, including UV–Vis spectroscopy, XRD, FTIR, SEM, TEM, DLS, zeta potential, and VSM analysis, to assess their structural, morphological, and magnetic properties. Results showed that fungus-mediated Fe3O4 nanoparticles were smaller, with an average size of 19.2 nm, and exhibited better crystallinity, surface functionalization, and colloidal stability than their commercial counterparts, which had an average size of 60 nm. Additionally, the fungal nanoparticles displayed superior magnetic properties with a saturation magnetization of 50 emu/g compared to 36 emu/g for commercial Fe3O4. The dye degradation potential of the nanoparticles was tested using methyl violet, methyl orange, and rose bengal dyes. Fungus-mediated Fe3O4 nanoparticles demonstrated higher dye removal efficiency than commercial Fe3O4, indicating enhanced catalytic activity due to their smaller size and larger surface area. This study highlights the potential of myco-synthesized Fe3O4 nanoparticles as effective agents for environmental remediation, particularly in removing of hazardous synthetic dyes from wastewater.

## 1 Introduction

Nanoparticles have received much interest because of their unique physicochemical features and applicability in various fields. Nanoparticles (NPs) are particles with different shapes and sizes ranging from 1 nm to 100 nm with specific properties that are based on their size, shape, content, and crystallinity that lead to high surface reactivity. Nowadays, nanoparticles are focused by researchers from different specialists, which have roles in different applications of the industrial, medical, and environmental sectors such as energy, agriculture, electronics, optics, catalysis, and remediation [[1], [2], [3], [4]].

**Fig. 1:**
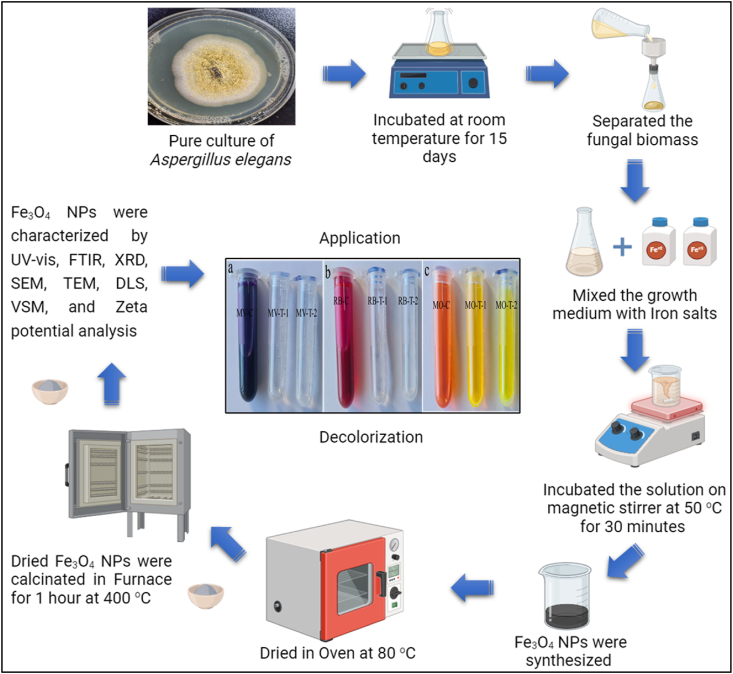
Preparation of fungal extract and biosynthesis of Fe3O4-NPs. The image was created with BioRender software.

To synthesize material within the range of the nanometer, there are two methods which are top-down and bottom-up. Based on the objects used as reducing agent, nanoparticle synthesis was divided into chemical, physical, and biological methods [5]. Chemical and physical methods can have adverse environmental effects due to chemicals, high temperatures, and pressures [6]. A more environmentally friendly approach is the biological method, using plant products and microorganisms. This approach is economically efficient, eco-friendly, and adaptable, eliminating the need for harmful chemicals, high pressure, and high temperatures. It's particularly suitable for creating nanoparticles for environmental cleanup (Fig. 1) [7].

Fungus - mediated nanoparticles, Myco-nanoparticle, Mycogenic nanoparticles, and myco-synthesized nanoparticles are the nanoparticles formed or fabricated by fungal components, while my-nanoparticle synthesis is the process or mechanism of formation of the nanoparticle using fungal components [8,9]. Moreover, the myco-nanoparticle synthesis can be broadly categorized into intracellular and extracellular processes to form the nanoparticles with different characteristics. The myco-nanoparticle synthesis involves intricate biological processes within fungi that reduce metal ions to form nanoparticles. On the other hand, fungi release a wide array of enzymes, proteins, or peptides that serve as reductive agents including naphthoquinones, anthraquinones, and nitrate reductase [[10], [11], [12]].

Generally, myconanotechnology is an exciting interdisciplinary field with various practical applications due to the diversity of the myco-nanoparticle characteristics [13]. Myco-nanoparticles are used in pathogen detection, wastewater purification, food preservation, nematicide production, and agricultural applications to boost crop yields and reduce the need for chemical pesticides [[14], [15], [16], [17], [18], [19]].

The motivation for this study stems from the need for more sustainable methods of producing nanoparticles, particularly in addressing environmental pollution. Synthetic dyes, which are frequently released into the water sources, are extremely stable and resistant to breakdown, and also, they are damage aquatic life and human health [20,21]. These pigments are removed by traditional wastewater treatment approaches using physical, chemical, or biological processes. However, chemical approaches can be expensive and produce hazardous byproducts, physical methods can result in secondary pollution, and biological methods are usually slow and ineffective [22].

Iron nanoparticles possess magnetic properties, making them suitable for medical and environmental remediation applications. *F*.*oxysporum* and *Verticillium* sp. release proteins that can enzymatically convert iron precursors into iron oxide, mainly in the form of magnetite (Fe_3_O_4_) at ambient temperature. The resulting nanoparticles have dimensions within 20–50 nm and 100–400 nm for *F*.*oxysporum* and *Verticillium* sp., respectively [11]. According to Saied et al. [23], the biomass filtrate from *A. niger* AH1 was employed to produce environmentally friendly Hematite (α-Fe_2_O_3_) nanoparticles, with an average size falling within the range of 58–76 nm as determined by DLS. The study aimed to synthesize the magnetite (Fe_3_O_4_) nanoparticles using an extract of *Aspergillus elegans* as a novel point of the study and perform the comparison between fungus-mediated Fe_3_O_4_ NPs and commercial Fe_3_O_4_ NPs in their properties and efficacy in the decolorizing synthetic dyes from water.

## 2 Materials and methods

### 2.1 Materials

The precursors used in the synthesis of magnetite nanoparticles include Iron Chloride Hexahydrate (FeCl_3_·6H_2_O) and Iron Chloride Tetrahydrate (FeCl_2_·4H_2_O) and were procured from a company named Biochem Chemopharma, France. Additionally, sodium hydroxide (NaOH) was also acquired from Biochem Chemopharma Company. Potato Dextrose Agar (PDA) medium (Accumix media, Microxpress factory, India). Methyl Violet (C_24_H_28_N_3_Cl), Rose Bengal (C_20_H_4_Cl_4_I_4_O_5_), and Methyl Orange (C_14_H_14_N_3_NaO_3_S) Dyes (Scharlab, Philippines).

### 2.2 Isolation and identification of Aspergillus elegans

**Fig. 2:**
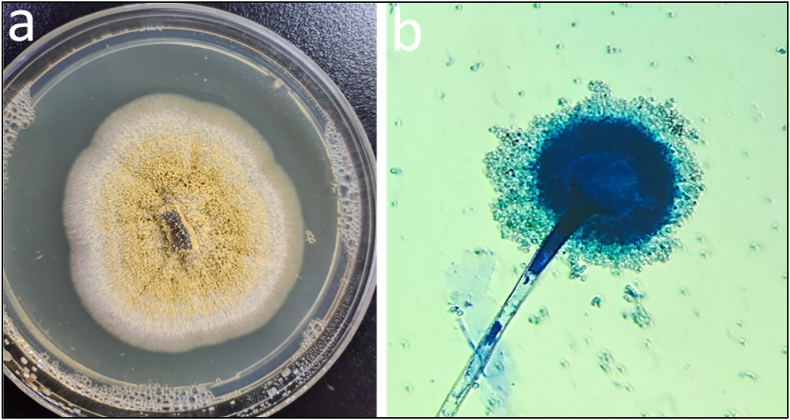
Morphological characterization of fungal species, a) Colony of *A.elegans* on PDA medium b) Sporangium and sporangiophore of *A.elegans* under a light microscope.

The soil samples were collected from the Soran municipal landfill (36.625146, 44.525702) to isolate *Aspergillus elegans*. The soil samples were diluted, and then a 100 μl sample from the diluted solutions was transferred onto PDA plates and incubated at 28 °C for 5 days. After incubation, single fungal colonies were sub-cultured onto fresh PDA plates to obtain pure cultures [24]. Identification of the isolated fungi was performed through morphological characterization of the fungal colonies and microscopic examination of fungal structures according to Zheng et al. [25], Muksy et al. [26], and Sidhoum et al. [27]. Light microscopy (model; Leica DM2700 P, Germany) was used to analyze the microscopic characterizations of the isolated fungi by performing methyl blue staining (Fig. 2).

### 2.3 Preparation of fungal extract

Fungal extract from *Aspergillus elegans* was prepared by culturing the fungi in a Potato Dextrose Broth (PDB) medium (200 ml of potato extract and 20g of glucose for each liter of distilled water), preserving the medium's pH about 7. The medium was then incubated at 28 °C for two weeks. Then, the culture medium was filtrated using Whatman filter paper no. 1. 100 ml of the filtrated medium solution was used to synthesize Fe_3_O_4_ NPs.

### 2.4 Myco-synthesis magnetite nanoparticle

In this work, magnetite nanoparticles were synthesized by performing a biological method using a fungal species, as shown in Fig. 1. The process was started by dissolving 2 g of FeCl_3_·6H_2_O and 1 gam of FeCl_2_·4H_2_O in 50 ml deionized water. Then, 100 ml of filtrated medium was added to the solution drop by drop. The mixture was constantly incubated at 50 °C while stirring for 30 min. Then, the mixture's color was turned to black, indicating that magnetite nanoparticles had been successfully produced.

Furthermore, 1N NaOH solution was added to the mixture to make pH 8–9. In addition, the solution was incubated in Oven at 80 °C until the water evaporated, which caused the nanoparticles to participate at the bottom of the container. To completely remove all impurities from the produced nanoparticles, the synthesized nanoparticles were then put through three rounds of washing (two with double-distilled water and one with ethanol). Following synthesis, the nanoparticles underwent an hour-long heat treatment in a furnace set at 400 °C in the calcination process.

### 2.5 Characterization of magnetite nanoparticles

A list of different methods and tools were used to characterize of the Fe3O4 nanoparticles generated via the myco-synthesis method. UV–Vis spectroscopy analysis is an important technique used to characterize the nanoparticle by measuring the light to detect the formation and stability of the nanoparticles [28]. X-ray diffraction (XRD) analysis was performed using a Panalytical X'Pert3 Powder instrument equipped with an XPERT-PRO diffractometer system and utilizing Cu Kα radiation. The diffraction data were collected over a 2θ range of 5°–70°, with a step size of 0.010° and a counting time of 0.5 s per step. The X-ray generator was set to 45 kV and 40 mA [29]. The obtained micrograph was compared with the Joint Committee on Powder Diffraction Standards (JCPDS) library to determine the crystalline structure of the nanoparticles. Additionally, the nanoparticle sample was lyophilized and subjected to further analysis. Morphological characteristics were investigated using scanning electron microscopy (SEM) with the FEI Model QUANTA 450. Zeta potential measurements were conducted with a Nano ZS90 Zeta sizer from Malvern Instruments, which featured a He–Ne laser operating at 633 nm with a power output of 5 mW. Dynamic light scattering (DLS) analysis was performed using the same Nano ZS90 Zeta sizer. The comprehensive analysis also included Vibrating-Sample Magnetometry (VSM), Transmission Electron Microscopy (TEM), and Fourier Transform Infrared (FTIR) spectroscopy.

### 2.6 Decolorization of the dyes by magnetite nanoparticles

A standard catalytic test reaction was performed on degraded dyes using Fungus - Mediated Fe_3_O_4_ NPs and Commercial Fe_3_O_4_ NPs. Three dyes were used in the experiment, including (Methyl violet Methyl orange, and Rose Bengal). The test was performed by adding 2 mL aqueous solution of the dyes (10 ppm), 50 μl of NaBH4 (0.1M), and 50 μl of nanoparticles (10 mg/ml) into a 10 ml tube. After a minute of reaction at room temperature, the solutions were centrifuged at 8000 rpm for 4 min to separate the nanoparticles from the solutions. Then, the solutions were read by the UV spectrophotometer (model; CE9500, Cecil Instruments) at 580 nm, Equation (1) was applied to calculate the dye removal efficiency according to Keyhanian et al. [30].

Eq (1) $$D\;\left(\%\right)=\frac{\left(A_{i}-A_{f}\right)}{A_{i}}\times 100\%$$

where A_i_ and A_f_ are the initial and final absorbance of the dye in the solution.

### 2.7 Statistical analysis

GraphPad Prism 8.0.2 was used to statistically analyze the data obtained from all experiments conducted in triplicate. An ANOVA test was performed to assess the significance of differences between treatments. P-values of less than 0.05 were considered statistically significant. The results are presented as mean ± standard error (SE).

## 3 Results and discussion

### 3.1 Isolation and identification of *Aspergillus elegans*

The results from the macroscopical and microscopical characterizations confirmed that the isolated fungus was *Aspergillus elegans* [24,25,31]. Fig. 2a exhibit the colony morphology of the *A. elegans*, as well as the microscopic features of its mycelium, sporangium, and conidia, are shown in Fig. 2b.

### 3.2 Characterization of fungus-mediated Fe_3_O_4_ nanoparticle

#### 3.2.1 UV–Vis spectroscopy analysis

**Fig. 3:**
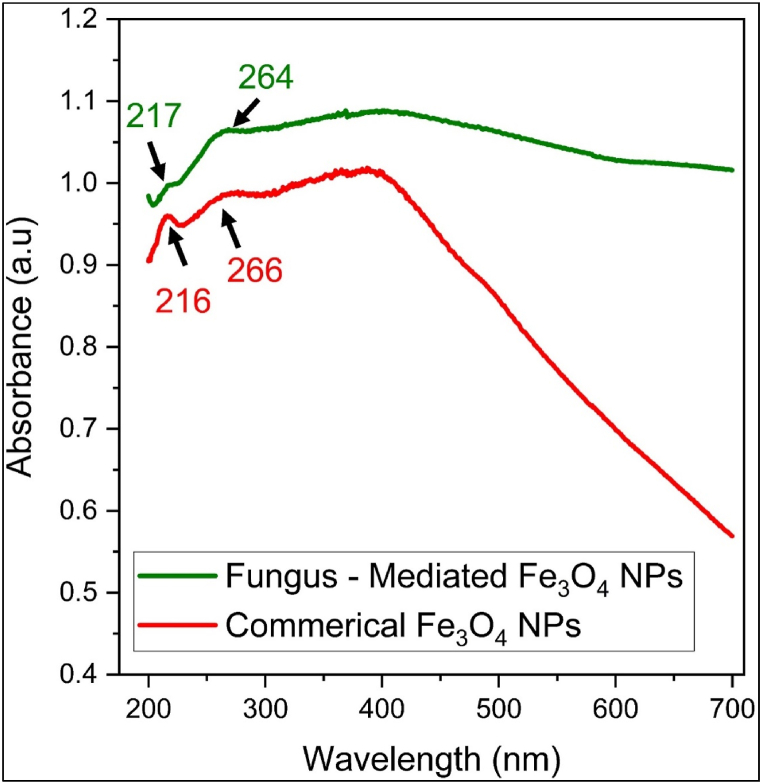
UV–Visible spectroscopic examination of fungus-mediated Fe_3_O_4_ NPs and commercial Fe_3_O_4_ NPs.

The UV–visible spectroscopic examination of Fe3O4 nanoparticles generated by fungal extract and commercial Fe3O4 nanoparticles showed distinct absorption peaks (Fig. 3). Commercial Fe_3_O_4_ displayed 216 and 266 nm, while fungus-mediated Fe_3_O_4_ displayed absorption peaks at 217 and 264 nm. The peaks shown by the study are fitting to the previous studies [[32], [33], [34]]. The marginally altered peak positions indicate variations in surface characteristics or particle size due to the synthesis techniques. Peaks at 264–266 nm belong to surface plasmon resonance, whereas those at 216–217 nm seem to be related to electronic transitions. These results suggest that Fe_3_O_4_ nanoparticles produced via fungal synthesis have slightly distinct electronic surroundings, which may have an impact on their uses in the fields of magnetic storage, medicinal applications, and catalysis [35].

**Fig. 4:**
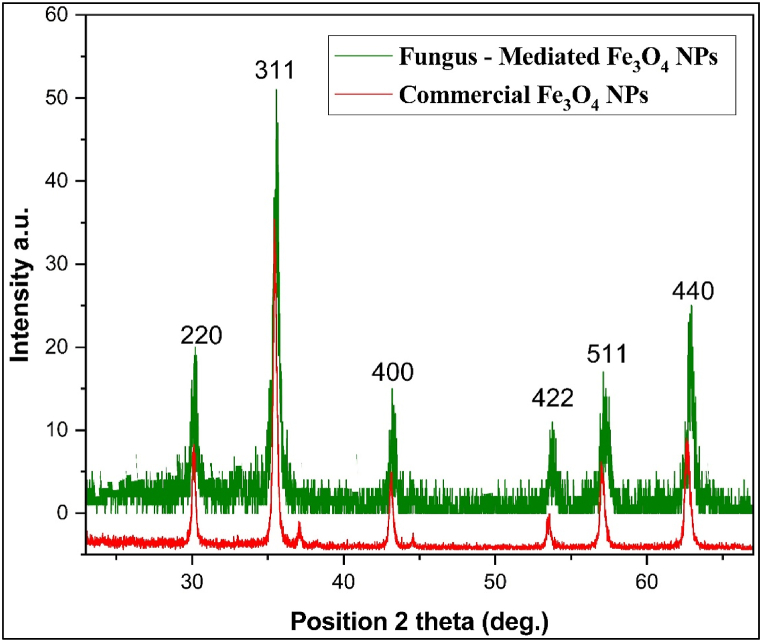
XRD patterns for fungus - mediated Fe_3_O_4_ NPs and commercial Fe_3_O_4_ NPs.

#### 3.2.2 XRD analysis

The XRD (X-ray Diffraction) patterns for both the Fe_3_O_4_ nanoparticles produced by fungal extract and the commercial Fe_3_O_4_ nanoparticles show characteristic peaks at approximately 30.2°, 35.56°, 43.19°, 53.76°, 57.17°, and 62.85° for the first, and 30.08°, 35.46°, 43.1°, 53.53°, 57.02°, and 62.7° for the second (see Fig. 4). The peaks line to the crystalline planes of magnetite's cubic spinel structure, indicating that the two synthesis processes generate Fe_3_O_4_ with the same phase and crystallographic structure. The slight variations in peak positions and strengths indicate minor differences in crystallite size and strain between the two samples. The stronger peaks identified in the fungal extract-mediated Fe_3_O_4_ demonstrate the improved crystallinity and probably a reduction in size due to more regulated nucleation and growth processes supported by the biomolecules in the fungus extract, which lead to enhanced magnetic characteristics [9,[36], [37], [38]].

#### 3.2.3 Fourier transform infrared (FTIR) analysis

**Fig. 5:**
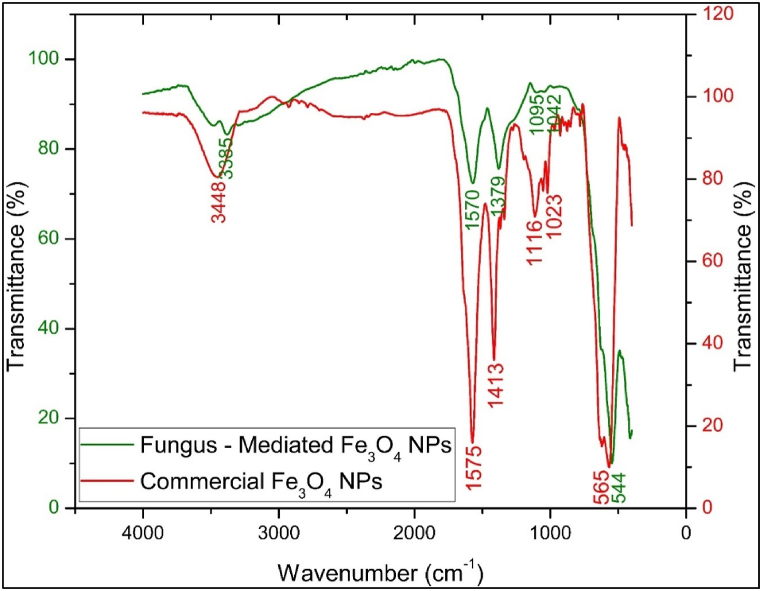
FTIR spectra of fungus - mediated Fe_3_O_4_ NPs and commercial Fe_3_O_4_ NPs.

The FTIR spectra comparison between fungus-mediated and commercial Fe_3_O_4_ nanoparticles reveals differences in their vibrational characteristics (Fig. 5). The OH- stretching vibration peak for fungus-mediated Fe_3_O_4_ NPs appears at 3348 cm-1, while for commercial Fe_3_O_4_ NPs, it is observed at 3390 cm-1, indicating a variation in surface chemistry and hydrogen bonding [[39], [40], [41], [42]]. The OH bending vibration peaks at 1575 cm-1 and 1565 cm-1 for fungus-mediated and commercial Fe_3_O_4_ NPs, respectively, suggest slight structural differences around the hydroxyl groups [43]. The CO stretching vibration peaks are detected at 1413 cm-1, 1116 cm-1, and 1023 cm-1 for fungus-mediated NPs, and at 1379 cm-1, 1100 cm-1, and 1036 cm-1 for commercial NPs, reflecting differences in surface functionalization and acid group interactions [44]. The Fe-O stretching vibration peak is prominent at 565 cm-1 for fungus-mediated NPs and at 544 cm-1 for commercial NPs, indicating variations in metal-oxygen bonding and crystalline environment [45]. Overall, the fungus-mediated Fe_3_O_4_ NPs exhibit more complex surface chemistry and a higher degree of surface functionalization than commercial NPs. The broader peaks and additional vibrational modes in fungus-mediated NPs suggest greater heterogeneity, likely due to the biological synthesis process, whereas commercial NPs display more uniform and well-defined peaks consistent with controlled chemical synthesis [46,47].

#### 3.2.4 Scanning electron microscope (SEM) analysis

**Fig. 6:**
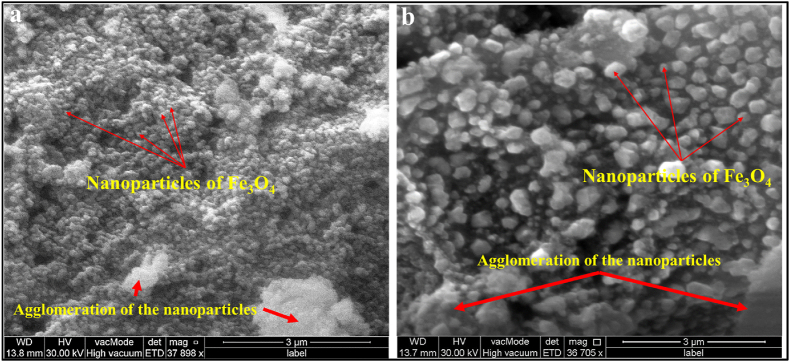
SEM images of the magnetite nanoparticle. a) fungus - mediated Fe_3_O_4_ NPs, b) commercial Fe_3_O_4_ NPs. The results showed an agglomerated structure in both magnetite nanoparticles.

SEM was used to describe the surface morphology of biosynthesized Fe_3_O_4_ NPs [48]. Fig. 6a and b shown the morphology of magnetite nanoparticle fungus–mediated and commercial Fe_3_O_4_ NPs, respectively. As shown, most of the Fe_3_O_4_ NPs formed during myco-synthesis are nanoscale and have a consistently hexagonal shape. Furthermore, the Fe_3_O_4_ NPs exhibit an agglomerated structure due to biological nanoparticle synthesis. Agglomeration or aggregation happens as a result of the strong attraction that biosynthetic NPs have for one another, contributing to the increased surface area of fungus–mediated Fe_3_O_4_ NPs [49]. Commercial magnetite nanoparticles have varied distributions and large sizes. Moreover, standard chemical procedures created magnetite nanoparticles that were strongly agglomerated [49,50].

#### 3.2.5 Transmission electron microscope (TEM) analysis

**Fig. 7:**
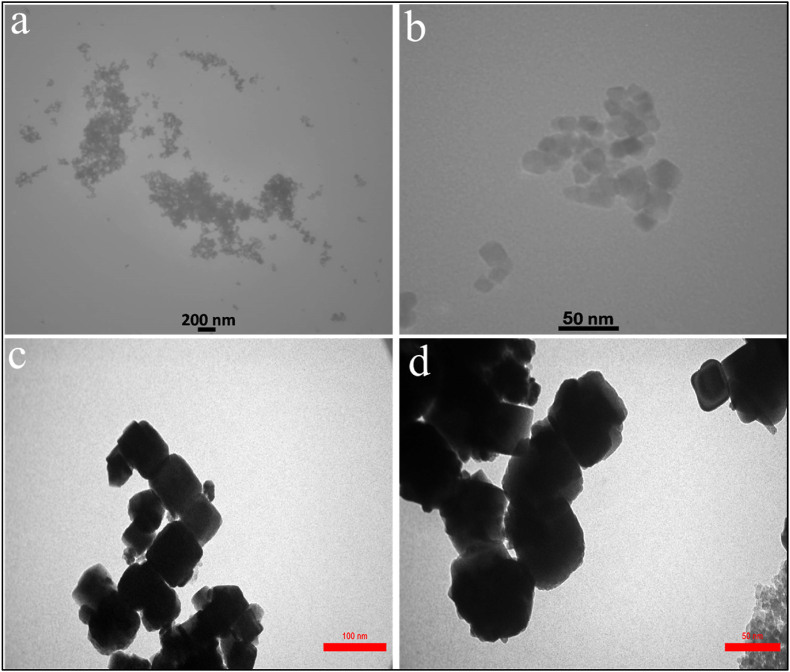
SEM images of the magnetite nanoparticle indicated both types of Fe_3_O4 NPs have hexagonal shapes. a and b) fungus - mediated Fe_3_O_4_ NPs, c and d) commercial Fe_3_O_4_ NPs.

**Fig. 8:**
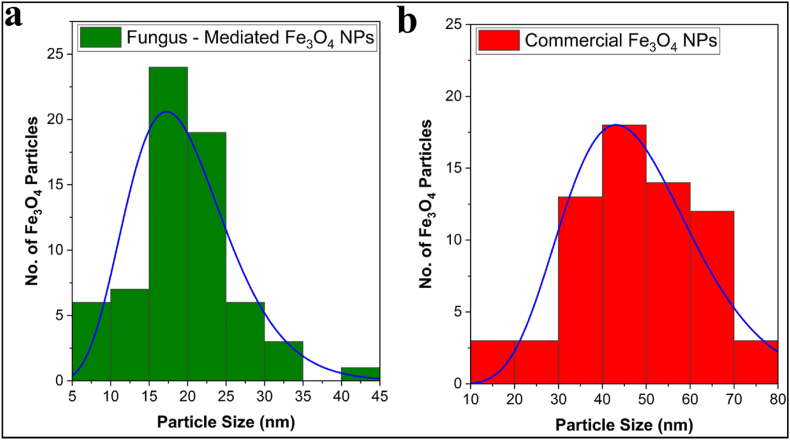
A particle size distribution histogram determined from the TEM images, a) fungus - mediated Fe_3_O_4_ NPs, b) commercial Fe_3_O_4_ NPs.

The shape and distribution of fungus-mediated and commercial Fe3O4 nanoparticles (NPs) were examined using transmission electron microscopy (TEM), which indicated both types of Fe_3_O_4_ NPs have hexagonal shapes Fig. 7ab and Fig. 7cd, respectively. The particle size histogram of the fungus–mediated Fe_3_O_4_ NPs revealed sizes ranging from 5 to 55 nm, with an average size of 19.2 nm (Fig. 8a). On the other hand, the commercial Fe_3_O_4_ NPs histogram (Fig. 8b) showed that most particle sizes were in the range of 60 nm–80 nm. These findings are consistent with earlier research [40,51]. The investigation shows that Fe_3_O_4_ NPs made via myco-synthesis are more stable and smaller than commercial nanoparticles. Smaller Fe_3_O_4_ NPs have a greater surface area, which means more capping agents are covering the fungus–mediated nanoparticles, which leads to improved nanoparticles' stability and zeta potential [52].

#### 3.2.6 Dynamic light scattering (DLS) analysis

**Fig. 9:**
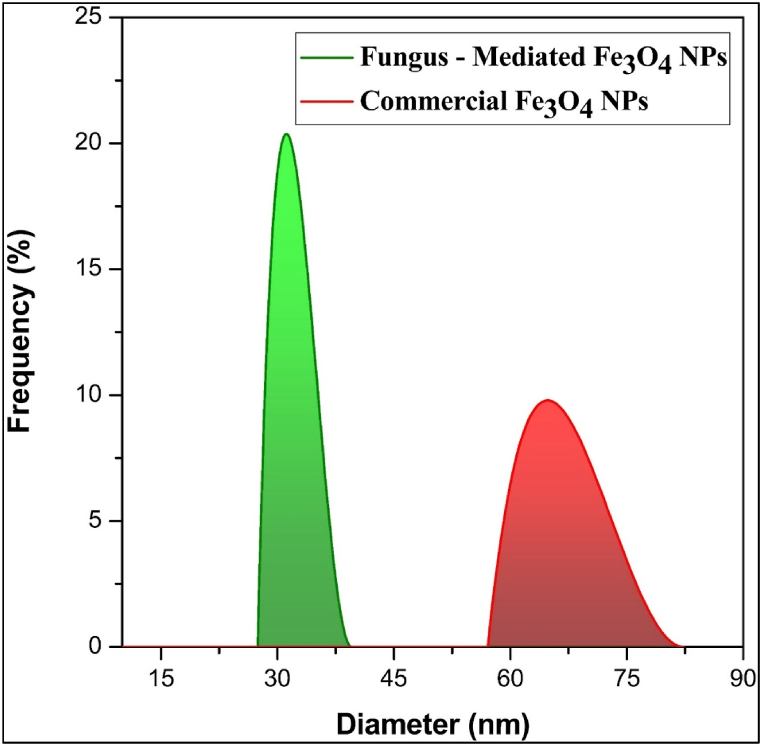
DLS analysis of magnetite nanoparticles determined difference in size of the both magnetite nanoparticles. a) fungus-mediated Fe_3_O_4_ NPs, b) commercial Fe_3_O_4_ NPs.

The DLS measurements have revealed different nanoparticle sizes resulting from different synthesis methods. The average size of commercial Fe_3_O_4_ NPs was 60 nm which was found to be much larger than fungus-mediated Fe_3_O_4_ NPs, their size was approximately 40 nm (Fig. 9). The use of fungus in biological synthesis most likely promoted the production of smaller nanoparticles due to specific biochemical compounds in the growth medium. On the other hand, the regulated nucleation and growth mechanisms employed in the chemical synthesis method produced bigger nanoparticles [53,54]. These results align with those of earlier research [40,51]. Using fungal extract in magnetite nanoparticle synthesis reduces particle size due to the bioactive compounds present. These compounds, including proteins, polysaccharides, and secondary metabolites, are reducing and capping agents. They facilitate controlled nucleation by reducing Fe³⁺ and Fe^2^⁺ ions, leading to smaller nanoparticles. The capping effect stabilizes the nanoparticles, limiting their growth and preventing agglomeration. Additionally, the slower growth kinetics associated with biological synthesis compared to chemical methods allows for better size control [55,56].

#### 3.2.7 Zeta potential analysis

**Fig. 10:**
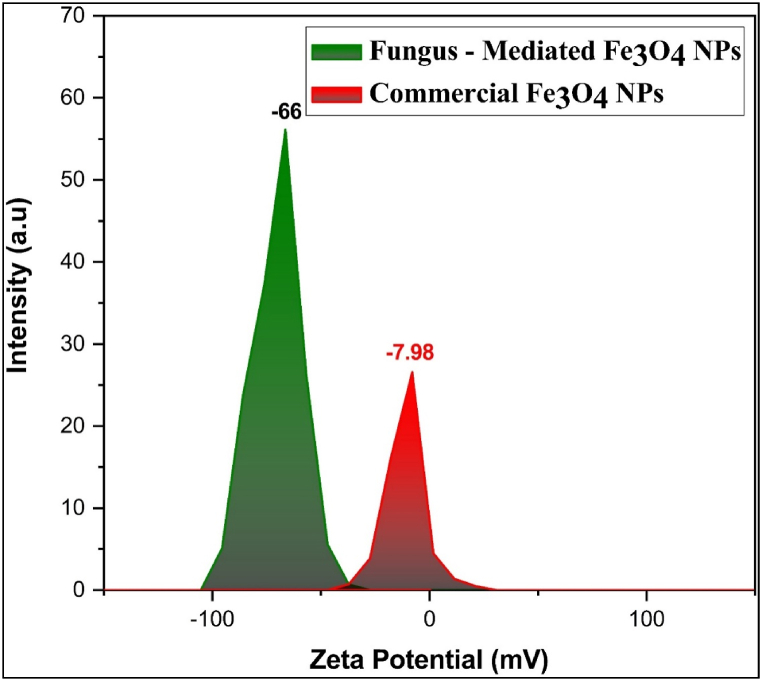
Zeta potential analysis shown large negative surface charge for both magnetite nanoparticles. a) fungus - mediated Fe_3_O_4_ NPs, b) commercial Fe_3_O_4_ NP.

The zeta potential results show a great difference in surface charge between the Fe_3_O_4_ NPs mediated by fungal extract (−66 mV) and commercial Fe_3_O_4_ nanoparticles (−7.98 mV) (Fig. 10). The fungus-mediated Fe_3_O_4_ has a strongly negative zeta potential, indicating greater colloidal stability due to increased electrostatic repulsion among particles, minimizing agglomeration. This could be related to the capping and stabilizing impact of proteins included in the fungal extract, which adsorb onto the nanoparticle surface and give a stronger negative charge [57]. Commercial Fe_3_O_4_ NPs have a lower negative zeta potential, making them less stable and more likely to assemble [58].

#### 3.2.8 Vibrating-sample magnetometer (VSM) analysis

**Fig. 11:**
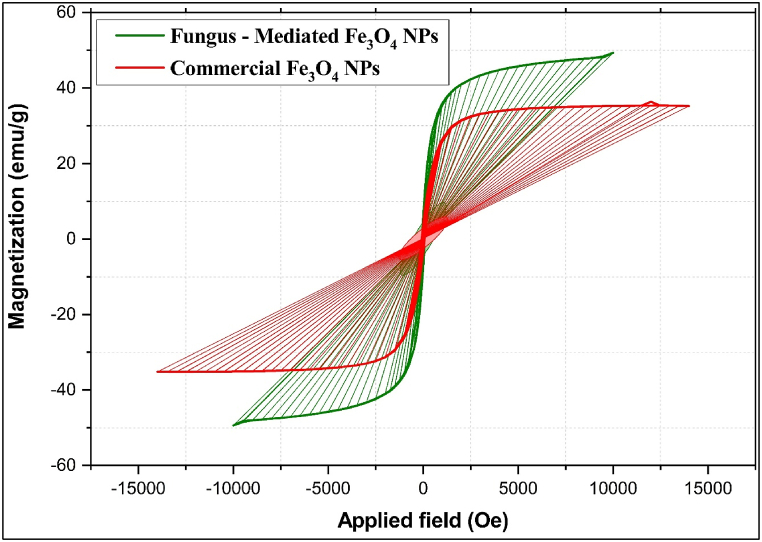
VSM investigations display the magnetic properties of magnetite nanoparticles. a) fungus-mediated Fe_3_O_4_ NPs, b) commercial Fe_3_O_4_ NP.

The VSM investigations display the magnetic properties of magnetite nanoparticles (Fig. 11). The Fe_3_O_4_ NPs mediated by fungal extract have a saturation magnetization of around 50 emu/g, which is much greater than the 36 emu/g observed for commercial Fe_3_O_4_ NPs [59,60]. This improved magnetic characteristic in fungal extract-mediated nanoparticles can be due to biomolecules, which may influence crystallinity and size distribution during the synthesis process, resulting in more uniform and smaller nanoparticles with a larger surface area. Smaller nanoparticles often have stronger saturation magnetization due to less surface spin disorder and better magnetic domain alignment [59,61]. Magnetite nanoparticles exhibit superparamagnetic behavior at smaller sizes, typically below 20–30 nm, due to the formation of single magnetic domains. All magnetic moments align uniformly in these tiny particles, preventing the multiple-domain formation seen in larger particles. Thermal fluctuations in small nanoparticles allow their magnetic moments to flip randomly, resulting in superparamagnetism. This means the nanoparticles display high magnetic susceptibility without retaining magnetization once the external magnetic field is removed. Superparamagnetic properties, arising from the small size, make these nanoparticles ideal for applications like magnetic resonance imaging (MRI) and targeted drug delivery, where controlled magnetic response is crucial [62].

#### 3.2.9 Decolorization of the dyes by magnetite nanoparticles

**Fig. 12:**
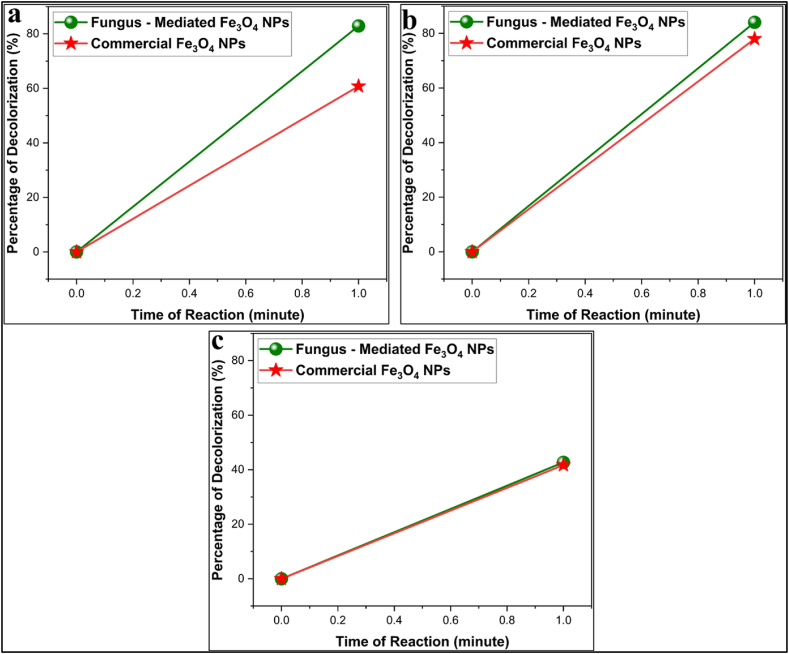
Percentages of decolorization efficiency of synthetic dyes with magnetite nanoparticles. a) Methyl Violet dye, b) Rose Bengal dye, c) Methyl Orange dye. The results show the high efficiency of fungus mediated Fe_3_O_4_ NPs compared to commercial Fe_3_O_4_ NPs. The experiment was done at room conditions. (For interpretation of the references to color in this figure legend, the reader is referred to the Web version of this article.)

The mean absorbance of the methyl violet, rose bengal, and methyl orange before and after being treated with commercial Fe_3_O_4_ NPs and fungus-mediated Fe_3_O_4_ NPs is shown in Table 1. Fig. 12a, b, and 12c shown the decolorization percentages of the methyl violet, rose bengal, and methyl orange, respectively. Compared to commercial Fe_3_O_4_ NPs, fungus-mediated Fe_3_O_4_ NPs demonstrated greater decolorization efficacy for all colors, especially methyl violet (82.9 %) and methyl orange (84.05 %) as shown in Fig. 12. Generally, the improved catalytic qualities and larger surface area of fungus-mediated Fe_3_O_4_ NPs lead to their high-performance [56].

**Table 1:** Absorbance (Mean±SE) of dyes treated with fungus-mediated Fe_3_O_4_ and commercial Fe_3_O_4_ NPs.

Treatments	Column1	Rose Bengal (550 nm)	Methyl Violet (580 nm)	Methyl Orange (465 nm)
**Control**	**Mean±SE**	0.2242 ± 0.001	0.2375 ± 0.0008	0.187 ± 0.0006
**Fungus - Mediated Fe₃O₄ NPs**	**Mean±SE**	0.03829 ± .0	0.03787 ± .0	0.1072 ± .0
	MD	0.18521	0.19963	0.0798
	*p* value	<0.0001	<0.0001	<0.0001
**Commercial Fe₃O₄ NPs**	**Mean±SE**	0.08798 ± .0	0.05225 ± .0	0.1091 ± .0
	MD	0.13622	0.18525	0.0779
	*p* value	<0.0001	<0.0001	<0.0001

**Fig. 13:**
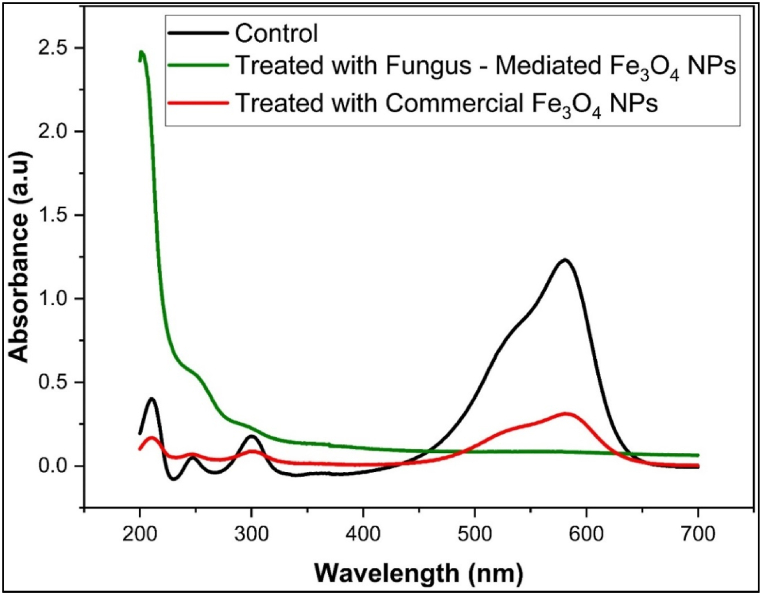
UV–Visible spectra of Methyl Violet dye solution before treatment (control) and after treated with fungus -mediated Fe_3_O_4_ NPs and commercial Fe_3_O_4_. (For interpretation of the references to color in this figure legend, the reader is referred to the Web version of this article.)

**Fig. 14:**
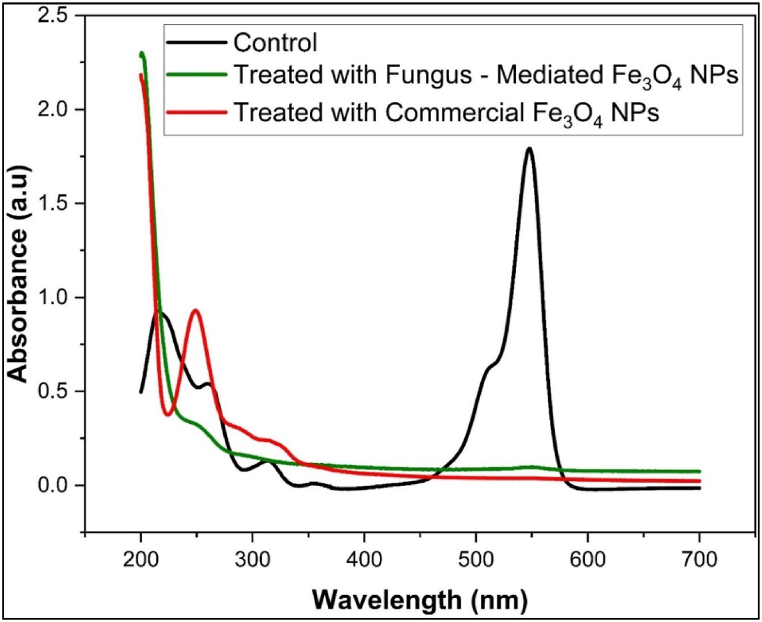
UV–Visible spectra of Rose Bengal dye solution before treatment (control) and after treated with fungus -mediated Fe_3_O_4_ NPs and commercial Fe_3_O_4_.

**Fig. 15:**
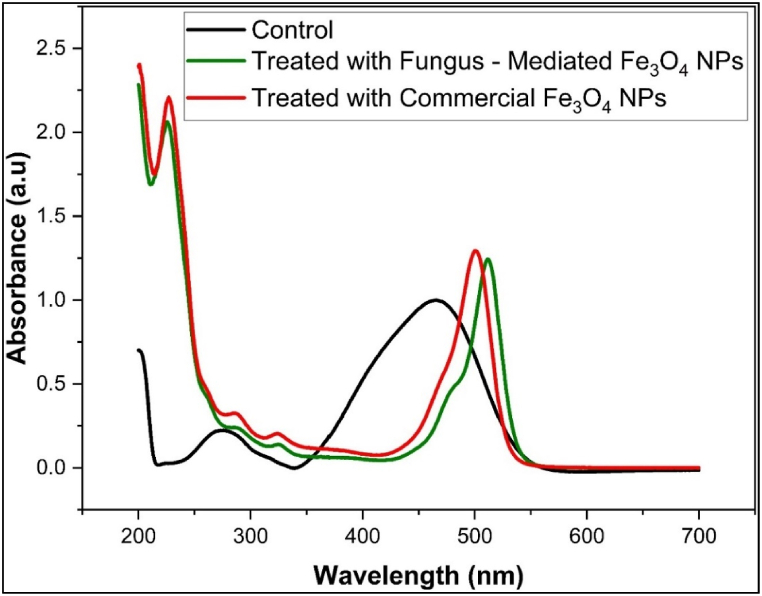
UV–visible spectra of Methyl Orange dye solution before treatment (control) and after treated with fungus-mediated Fe_3_O_4_ NPs and commercial Fe_3_O_4_. (For interpretation of the references to color in this figure legend, the reader is referred to the Web version of this article.)

UV–Visible spectroscopy was used to confirm the degradation of methyl violet, methyl orange, and Rose Bengal dyes after treatment with fungus-mediated and commercial Fe_3_O_4_ nanoparticles compared with untreated dyes (Controls). The initial absorption peaks of methyl violet were seen at 580 nm (1.3 a.u.), 300 nm (0.25 a.u.), 250 nm (0.12 a.u.), and 220 nm (0.47 a.u.) (Fig. 13). Post-treatment with fungus-mediated Fe_3_O_4_ resulted in the near-total breakdown, possibly due to improved surface characteristics and catalytic activity driven by bioactive chemicals from fungi. While commercial Fe_3_O_4_ nanoparticles show peaks with a lower intensity that indicate partial degradation. While the initial peaks of Rose Bengal dye were observed at 550 nm (1.79 a.u.), 320 nm (0.125 a.u.), 260 nm (0.54 a.u.), and 215 nm (0.927 a.u.) (Fig. 14). Following the application of fungus-mediated Fe_3_O_4_ treatment, no peaks were observed, signifying total breakdown. While, after using commercial Fe_3_O_4_ treatment, residual peaks were seen at 320 nm (0.225 a.u.) and 250 nm (0.91 a.u.), suggesting partial deterioration. However, the UV–visible spectroscopy showed main peaks for methyl orange at 465 nm and 275 nm before being treated with a nanoparticle as its structure (Fig. 15). After the nanoparticles were used to treat the dye, the peaks were shifted, and new peaks were observed from both treatments at 225 nm, 285 nm, 315 nm, and 500–510 nm, indicating partial degradation and intermediate product formation [63].

This lower efficacy of the commercial Fe_3_O_4_ nanoparticle could be due to less optimal surface properties and the absence of bioactive enhancements, whereas fungus-mediated Fe_3_O_4_ benefits from a higher surface area, smaller particle size, and more active catalytic sites [[64], [65], [66]]. Generally, these properties of fungus-mediated Fe_3_O_4_ facilitate more effective interaction and decomposition of dye molecules, making fungus-mediated Fe_3_O_4_ a more efficient catalyst for dye degradation compared to its commercial counterpart and the magnetite nanoparticle synthesized by fungal extract have higher magnetic field as shown by VSM analysis which leads to be an excellent method for water remediation due to separate easily from the treated water [67].

**Fig. 16:**
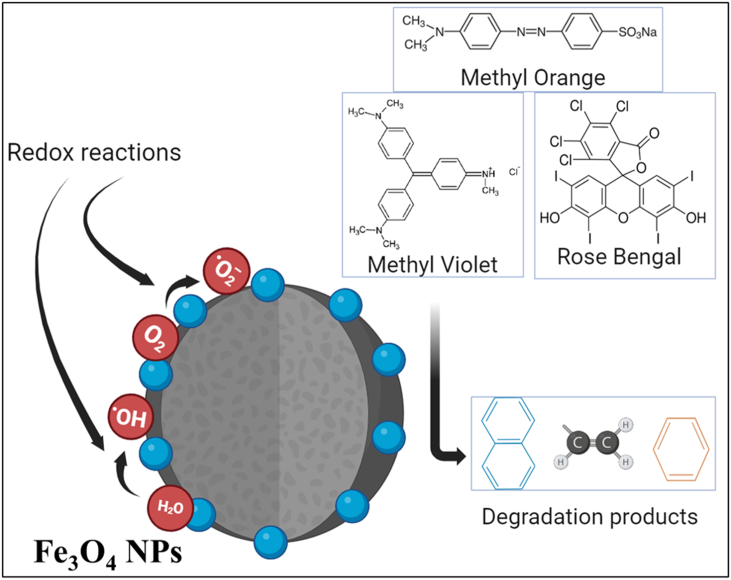
General mechanism of catalytic activity of Fe_3_O_4_ NPs to degrade the dyes into different chemical compounds as byproducts of its redox reactions.

Decolorization mechanism of dyes by magnetite nanoparticles involves several key processes, primarily adsorption and catalytic degradation (Fig. 16). Magnetite nanoparticles exhibit a high surface area and can effectively adsorb dye molecules onto their surfaces due to their magnetic properties and functional groups. Once adsorbed, the presence of reactive species, such as hydroxyl radicals generated through redox reactions that can facilitate the breakdown of the dye molecules. The catalytic activity of magnetite enhances the electron transfer processes, leading to the cleavage of chromophoric bonds in the dye structure, and decolorization. Additionally, the magnetic properties of magnetite allow for easy recovery and reuse of the nanoparticles, making this method not only efficient but also environmentally friendly for wastewater treatment applications [68,69].

## 4 Conclusions

In conclusion, the study shows that Aspergillus elegans extracts can effectively biosynthesize magnetite nanoparticles, and they are better than commercially manufactured Fe_3_O_4_ NPs in terms of size, stability, and decolorizing synthetic dyes. The biogenic Fe_3_O_4_ NPs had improved crystallinity, stability, and size, as evidenced by UV–Vis spectroscopy, XRD, FTIR, SEM, TEM, DLS, Zeta potential, and VSM. The myco-synthesized nanoparticles demonstrated much greater decolorization rates for methyl violet, methyl orange, and rose bengal dyes, showing superior catalytic efficiency. This environmentally friendly methodology not only provides a sustainable way for nanoparticle synthesis, but also an efficient wastewater treatment solution. The findings highlight the potential of myco-nanotechnology in environmental remediation, which promotes safer and cleaner water resources. The study recommends that the applicability of the synthesis technique to a wider spectrum of pollutants and its scalability should be examined in future research.

## CRediT authorship contribution statement

**Azhin H. Mohammed:** Writing – original draft, Visualization, Validation, Supervision, Investigation, Funding acquisition, Conceptualization. **Renjbar M. Mhammedsharif:** Writing – review & editing, Writing – original draft, Visualization, Software, Resources, Project administration, Methodology, Investigation, Formal analysis. **Parwin J. Jalil:** Writing – review & editing, Visualization, Validation, Software, Methodology, Investigation, Formal analysis. **Sida M. Mhammedsharif:** Visualization, Software. **Ahmed S. Mohammed:** Writing – review & editing, Supervision, Project administration.

## Availability of data and materials

The data supporting the conclusions of this article are included in the article.

## Ethics approval and consent to participate

Not applicable.

## Funding

This work had no funding.

## Declaration of competing interest

The authors declare that they have no known competing financial interests or personal relationships that could have appeared to influence the work reported in this paper.
